# Effect of Dietary Ginger (*Zingiber officinale* Roscoe) and Multi-Strain Probiotic on Growth and Carcass Traits, Blood Biochemistry, Immune Responses and Intestinal Microflora in Broiler Chickens

**DOI:** 10.3390/ani8070117

**Published:** 2018-07-14

**Authors:** Mehdi Qorbanpour, Taha Fahim, Faramin Javandel, Mehran Nosrati, Erwin Paz, Alireza Seidavi, Marco Ragni, Vito Laudadio, Vincenzo Tufarelli

**Affiliations:** 1 Department of Animal Science, Rasht Branch, Islamic Azad University, Rasht 14536, Iran; mahdigh1342@gmail.com (M.Q.); taha.fahim1987@gmail.com (T.F.); fdjavandel@yahoo.com (F.J.); nosrati@iaurasht.ac.ir (M.N.); alirezaseidavi@iaurasht.ac.ir (A.S.); 2 Institute of Agriculture, University of Western Australia, Crawley, WA 6009, Australia; e.paz01@ufromail.cl; 3 Departamento de Producción Agropecuaria, Universidad de La Frontera, Temuco 01145, Chile; 4 Department of Agricultural and Environmental Science, University of Bari ‘Aldo Moro’, Bari 70125, Italy; marco.ragni@uniba.it; 5 Department of DETO, Section of Veterinary Science and Animal Production, University of Bari ‘Aldo Moro’, Valenzano, Bari 70010, Italy; vito.laudadio@uniba.it

**Keywords:** medicinal plants, broiler, growth, blood parameters, immune system, intestinal microflora

## Abstract

**Simple Summary:**

In the last decade, there has been growing interest in the use of natural herbs and probiotics as alternatives to antibiotics in feeds to improve animal productivity and to maximize their potential output. Therefore, the objective of this study was to determine the effect of different levels of ginger powder and a commercial multi-strain probiotic in the diet on broiler performance, immune response, microbiota, haematology and carcass characteristics. Based on findings, dietary supplementation with both ginger or probiotics showed significant influence on birds’ immune response, probably because ginger had strong antioxidant activity and the probiotics stimulated the production of natural antibodies.

**Abstract:**

A total of 225 day-old male broiler chicks (Ross-308) were randomly allocated to five treatment groups, with three replicates in a completely randomized design for 42 days. Birds were fed a basal-diet supplemented with: no additive (control-diet), multi-strain probiotic (*Lactobacillus acidophilus, Lactobacillus casei, Enterococcus faecium* and *Bifidobacterium thermophilum*), or 0.15, 0.20 and 0.25% ginger (*Z. officinale*) powder, respectively. The results show no significant differences among treatments for growth traits and carcass characteristics, whereas using probiotics and ginger at all levels resulted in a significant decrease of gizzard weight and abdominal fat compared to the control group. Dietary treatments did not affect blood biochemistry and antibody production against sheep red blood cells (SRBC), IgG and IgM; however, antibody titre was higher in birds fed 0.25% ginger than other diets after 7 days post injection. The *Lactobacillus* counts in ileal content of birds fed 0.20 and 0.25% ginger were higher compared to the other treatments. In conclusion, dietary supplementation with either ginger or probiotics showed a significant influence on birds’ immune response, probably because ginger had strong antioxidant activity and the probiotics stimulated the production of natural antibodies.

## 1. Introduction

Recently, there has been an interest in improving poultry health by using environmentally-friendly products [1]. Commonly, under intensive production systems chickens are stressed mainly due to sudden changes in the environments (chilling or overheating), parasites, transportation and vaccination [2]. The combination of these factors negatively affects productive efficiency and carcass characteristics promoting permanent changes in intestinal microbiota and leading to immune system disequilibrium [3].

Antibiotics and synthetic agents generally used as feed additives in livestock are controversial due to their potential toxicological effects. Moreover, there is an increasing trend for natural products in order to reduce the use of synthetic substances [2] Thus, medicinal plants and probiotics are currently considered safe and offer a possible alternative to satisfy customer demands and current market challenges [1,4].

*Zingiber officinale* Roscoe, belonging to the Zingiberaceae family, popularly known as ginger, is a monocotyledonous herbaceous plant and one of the most common food-flavouring spices used worldwide [5]. In recent years, several pharmacological properties of ginger, such as antinflammatory, analgesic, gastrointestinal regulating agent, antioxidant and antimicrobial properties have been identified [6]. Live microbial feed preparations such as probiotics, prebiotics, or synbiotics play an important role in increasing the resistance to disease by improved immune response, thereby reducing the use of antibiotics [7,8]. Some probiotic microorganisms have been reported to produce different types of bacteriocins, organic acids and reuterin which act by preventing pathogen growth [9]. Furthermore, probiotics supplements can affect the intestinal environment by increasing desirable microbial growth [8]. In parallel, there are few studies evaluating the performance and health-related traits of broilers receiving either ginger (*Z. officinale*) or particular mixtures of probiotics cultures to verify the possible beneficial effects of natural feed additives as substitutes of probiotics.

Therefore, the objective of this study was to determine the effect of different levels of ginger powder and a commercial multi-strain probiotic in diet on broilers performance, immune response, microbiota, haematology and carcass characteristics.

## 2. Materials and Methods

### 2.1. Animals and Experimental Diets

This experiment was performed in a poultry farm in Abkenar, Guilan, Iran, and at the Islamic Azad University, Rasht Branch, Iran. Procedures with animals were performed following good veterinary practice for animal welfare according to the national laws in force and in accordance with the Declaration of Helsinki, and the protocol was approved by the Ethics Committee of the Islamic Azad University, Iran (no. 93/10-14). The facilities were carefully prepared and sanitized before arrival of the birds. Over the next 48 h the facilities remained closed and the ventilation system was turned on 24 h before the beginning of the trial. A total of 225 day-old male chicks of the Ross 308 strain (Aviagen) were randomly allocated to five treatment groups, with three replicates in a completely randomized design for 42 days. The birds were housed in pens having 15 birds/pen (2.0 × 1.0 m). Feeders and drinkers were washed and cleaned daily, and water was provided ad libitum. Animals were fed a balance diet according to the producer’s recommendations (Aviagen). Birds were maintained with heater following the temperature program based on instructions for Ross 308 broilers (Aviagen). Air humidity was kept at 60–70% in the 1st week of age and at 50–60% in the 2nd–6th weeks of age by spraying water on the floor. The 100-watt lamps were mounted at a height of 2.0 m from the floor. The artificial lighting program was 23 h of light followed by 1 h in darkness until day 42. Sanitation procedures and health practices were effective throughout the study. The birds were vaccinated against bronchitis disease (1st and 18th days of age), Newcastle disease (1st and 18th day of age), influenza disease (1st day of age) and Gumboro disease (14th and 24th day of age). The ingredients and nutrient composition of diets fed during the starter (1–21 days of age) and finisher (22–42 days of age) periods are reported in Table 1.

The multi-strain probiotic (PrimaLac) fed to broilers was added as a lyophilized mixture containing 1 × 10^8^ CFU/g of *Lactobacillus casei*, *Lactobacillus acidophilus*, *Bifidobacterium thermophilum*, and *Enterococcus faecium*. The ginger (*Z. officinale* Roscoe) was added to the diet as a powder. The dietary treatments fed to broilers for 42 days were as follows:

Treatment 1: control diet (without any additive); *Treatment 2*: diet including probiotics (following the recommended level of manufacturer’s instructions: 0.900 g/kg from 1–14 days, 0.454 g/kg from 15–28 days, 0.225 g/kg from 29–42 days); *Treatment 3*: diet including ginger at 0.15%; *Treatment 4*: diet including ginger at 0.20%; and *Treatment 5*: diet including ginger at 0.25%.

### 2.2. Performance and Carcass Traits

Feed intake and body weight of broilers were weekly recorded. Feed conversion ratio was calculated based on conventional protocol. At 42 days and after 4 h of fasting, one bird from each replicate was selected and killed by cervical dislocation and then immediately bled, and used to assess carcass yield, distribution of meat and gastrointestinal tract characteristics. Birds were fully plucked by the dry-pecking method. Feet were separated from the carcass at the tibio-tarsal joint. In order to calculate carcass weight, neck, wingtips, gut and liver were removed.

### 2.3. Haematology Analysis

Before blood sample collections, feed was removed from all birds for a period of 4 h in an attempt to allow stabilization of blood constituents. On the morning of day 4, a 2.5 mL of venous blood sample was collected from the ulnaris vein of one bird from each treatment into ethylene diamine triacetic acid (EDTA) tubes. Blood samples were centrifuged at 3000 rpm for 20 m to assure separation of the blood cells and then stored at −20 °C for further analysis. The cholesterol and triglyceride levels were determined enzymatically (TeifAzmoon Pars, Co., Tehran, Iran). Estimation of serum cholesterol in blood samples was assessed by cholesterol oxidase procedure based on calorimetric reactions. Plasma triglycerides were completely hydrolysed to release glycerol and quantified spectrophotometrically [10]. The concentrations of high-density lipoprotein (HDL) and low-density lipoprotein (LDL) were measured by analytic kits (TeifAzmoon Pars Co, Tehran, Iran). Glucose oxidase kit (Teif Azmoon Pars, Co., Tehran, Iran) and uric acid-uricase enzyme kits (Teif Azmoon Pars, Co., Tehran, Iran) were used to determine plasma glucose and plasma uric acid, respectively.

### 2.4. Immunity Traits

In order to evaluate the humoral immunity response of broilers against Newcastle disease virus (NDV) and avian influenza (AI) by hemagglutination-inhibition test [11], chicks (3 birds per treatment) were injected intramuscularly with 0.5 mL of 5% suspension sheep red blood cells (SRBC) diluted in phosphate-buffered saline (PBS) at 21 and 35 days, respectively and then blood samples were retrieved from the brachial veins of 1 bird per pen (3 birds per treatment) 7 days later on days 28 and 42, respectively. The hemagglutination assay test was used to determine the total antibody response against SRBC as described by Van Heugten and Spears [12]. Serial two-fold dilutions of serum were prepared at 56 °C for 30 min in order to inactivate the complement. The PBS (0.01 M; pH 7.4) was dispensed into 96-well U-bottom microtiter plates, then was added 50 µL of serum samples and 50 µL of 1% SRBC in PBS into each well. Antibodies of immunoglobulin G (IgG) were measured using 1.4% 2- mercaptoethanol. For the determination of immunoglobulin M (IgM) titers, a measure of the difference between total antibody and IgG was done. The titres of the antibody were expressed as log_2_ of the highest dilution of serum that agglutinated an equal volume of a 0.5% SRBC suspension in PBS.

### 2.5. Microbiota Analysis

On day 42, two chickens were separated from each experimental group and sacrificed. The contents of ileum section were collected for microbial culture. Tubes were weighed, autoclaved and caecal contents added to Petri dishes. The MRS agar (Man Rogosa Sharpe agar, 1.10660.500) was used to culture Lactobacilli, and nutrient agar (1.05450.0500) was used to culture total aerobic bacteria, Eosin Metilan-Blou (EMB, 1.01347.0500) was used for *Escherichia coli*, and MacConkey agar (105465.0500) was used for coliforms, respectively. After shaking for 30 min, 1 mL samples of the suspension was added into 9 mL PBS for serial dilution at 10^−2^, 10^−3^, 10^4^, 10^−5^ and 10^−6^; then, 100 μL was removed from the 10^−4^, 10^−5^ and 10^−6^ dilutions and added to the Petri dishes, which were incubated under anaerobic conditions at 37 °C for 72 h. Bacterial units were counted by a colony counter and adjusted to 1 g sample.

### 2.6. Statistical Analysis

Data were analyzed as a completely randomized design including five treatments and subjected to statistical analysis using the general linear model procedures of the SPSS 11.0 (SPSS Inc., Chicago, IL, USA). Differences among means were assessed using Duncan’s multiple range test. Significance level was set at *p* < 0.05.

## 3. Results

All chickens maintained good health status during the experiment and no mortality was recorded (data not shown). A significant effect on feed intake between treatments was only observed at the starter period of age (days 1–21) (Table 2). In addition, weight gain and feed conversion ratio showed differences among dietary treatments during the starter period of age but it was not significant. Overall, the findings showed that the experimental diets did not affect (*p* > 0.05) the daily feed intake, weight gain and feed conversion ratio throughout the whole treatment period.

The effect of the experimental diets on broiler carcass measurements are reported in Table 3. The weights related to the different components, such as carcass, cookable carcass, breast, thighs, wings, and liver were not significantly influenced by dietary treatments. Birds receiving supplemented diets had significant lower gizzard weight and abdominal fat compared to the control group (*p* < 0.05). It was also observed that the abdominal fat weight in the group receiving 0.25% of ginger was lower compared to the other treatments (*p* < 0.05). The Table 4 summarizes the influence of diets on broiler serum biochemistry at day 42 of age. None of the serum biochemical parameters were significantly affected by the treatments among the different groups. However, HDL concentrations were numerically higher and LDL lowers in animals receiving 0.25% ginger (*p* > 0.05). There were no significant differences in serum levels of total cholesterol and triglycerides.

The effects of dietary treatments on birds immune response are shown in Table 5. The antibody titers against NDV and AI disease at 28 and 42 days of age were not significant among treatments; furthermore, no significant responses to inoculation with SRBC after the first and second injections were observed among experimental treatments. Despite this, birds receiving 0.25% ginger had the highest (*p* < 0.05) antibody production after 7 days from the first inoculation, while birds in the probiotics group had the highest antibody production at day 42 (*p* > 0.05). After the first vaccination, the antibody response to NDV and AI was higher in the probiotics group, but the difference was not significant. The measurements of bacteria in the ileum did not differ significantly between treatments (Table 6). The control group showed the highest count of total aerobic bacteria than the other treatments, whereas the *Lactobacillus* count was higher in birds fed with 0.2 and 0.25% ginger (*p* < 0.05).

## 4. Discussion

The present report provides detailed evidence on the influence of ginger at incremental inclusion rates, dose-response and probiotics on growth performance, serum biochemistry, immunity and ileum microbiota in broilers. The results of this study demonstrated that dietary supplementation of ginger and probiotics did not affect the weight gain and feed efficiency of broilers. Zomrawi et al. [13] concluded that ginger root powder at levels of 0.5% and 1% in broiler diet had no significant effect in feed conversion ratio, decreasing also the weight gain. The inclusion of ginger in the diet reduced the abdominal fat and gizzard weight in a dose-dependent manner, revealing the greatest weight loss when birds were fed 0.25% ginger. These findings are consistent with the previous findings of Ademola et al. [14] who reported that dietary supplementation with ginger in broilers reduced the relative weight of abdominal fat. Moreover, Abdullah [15] demonstrated the hypoglycaemic, hypocholesterolemic and hypolipidemic potential of ginger when fed to rats. However, we did not observe any differences in biochemical parameters of broilers fed the different diets.

The feed intake of broilers was affected principally during the first period of age (1–21 days; *p* < 0.05), while no differences were found throughout the whole growth cycle; this result was due probably to gut microbiota in young birds not yet being stable and not still being well developed.

Probiotics are a group of beneficial microorganisms affecting the host response by restoring proper intestinal flora. The environment of gut micro-ecology is likely affected by daily probiotics intake preventing the development of harmful microorganisms [16]. Several studies suggested a possible beneficial role of probiotics consumption on broiler performance [3,9,16], while others reports concluded that probiotics did not influence poultry performance [7,17]. The effectiveness of probiotics is mainly determined by species composition, administration level, frequency of application and environmental stress factors [18].

In our study, the experimental diets significantly decreased birds’ gizzard weight at the end of the feeding trial, whereas the rest of the internal organs were not affected by experimental diets. Detectable irregularities in the internal organ weights are a sensitive index to reduce anti-nutritional factors after exposure to toxic substance [1,19].

The dietary treatments did not affect the serum biochemistry of broilers, despite birds receiving 0.25% ginger having a tendency to modify HDL and LDL concentrations. Alizadeh-Navaei et al. [20] established that the supplementation with ginger powder significantly decreased the levels of triglycerides, total cholesterol, LDL and very-low density lipoprotein (VLDL). Furthermore, Hong et al. [21] reported in broiler chickens receiving ginger essential oil higher HDL and lower VLDL levels, whereas no significant difference was observed on LDL concentration. In general, normal levels of blood protein and glucose indicate adequate nutritional status and normal systemic protein function, while hypertriglyceridemia, low HDL levels and high VLDL triglyceride usually result in metabolic syndrome [14,21].

Experimental diets containing either probiotics or plant extract had effects on immune response of broilers. Conversely, we expected a possible immune evidence according to previous studies, but our results show that the composition of diet could possibly modulate the different antibody response. According to Haghighi et al. [22], the administration of *Lactobacillus acidophilus* and *Lactobacillus casei* improved the IgA response and did not directly affect the IgG production. In addition, broilers’ feed including different *Lactobacilli* species showed higher IgM and IgG titres [23]. In overall, the experimental diets showed an increase in immune-related parameters including IgM, IgG and IgT compared to the control group, but these differences were not significant. The probiotic treatment did not affect significantly the antibody response to NDV or AI vaccination, although, in general, an increase in antibody production at day 28 was found compared to day 42. Stimulation of natural antibodies by probiotics could potentiate the systemic antibody response reducing the colonization of intestinal pathogens [7,22]. Recently, it has been shown that ginger extract more than enhances the serological response and had an antioxidant activity (both in vivo and in vitro) mainly due to pungent active principles such as gingerols and shogaols [6]. In addition, it has been reported to be associated with a strong anti-inflammatory and anti-apoptotic activity [4]. According to the results obtained by Toghyani et al. [24], broilers response to NDV vaccine was not affected by the inclusion either of 1 or 2% of garlic powder.

Generally, the type and total number of bacteria present in the gut of chickens can vary from one individual to another depending on the feed, physiological state and rearing conditions. The conventional microbial flora of chickens is naturally colonized by various species of *Lactobacillus* [25]. Furthermore, the specific mode of action of probiotics is not still clear due to dependence upon the nature of the culture and also due to the difference among species. According to Lan et al. [3], probiotic *Lactobacillus* strains re-established the microbial community structure by maintaining the natural equilibrium of native bacterial microbiota. Plants can produce a wide variety of antimicrobial agents derived from their specific bioactive components, mostly secondary metabolites. Some plants including ginger exerted a weak antimicrobial activity [26]. Ginger extract showed in vitro antibacterial activity against *Pseudomonas aeruginosa, Salmonella typhimurium. Escherichia coli and Candida albicans* [27]. However, our findings showed that intestinal microbiota was not significantly altered by experimental diets with either probiotics or ginger, indicating a healthy microflora. Moreover, a number of conflicting bacteria in the gut of broilers may determine a reduction of feed efficiency and animal performance [28].

## 5. Conclusions

Ginger and probiotics are considered safe due to having no acute toxic side effects as reported through the experimental period. According to our results, the experimental diets decreased gizzard weight and abdominal fat, but there were no effects in broiler growth performance and carcass characteristics. We did not find any significant effect on serum biochemistry, but there was a trend towards healthier profiles in broilers receiving experimental diets. It can be also concluded that dietary supplementation with either ginger and probiotics showed an influence on immune response of broilers, probably because ginger had strong antioxidant activity, whereas microbial feed supplement stimulated natural antibodies production.

## Figures and Tables

**Table 1:** The ingredients and nutritive contents of the basal-diet.

Ingredient (%)	Starter (1–21 Days)	Finisher (22–42 Days)
Corn	56.9	58.7
Soybean meal (43% CP)	33.1	30
Fish meal	3.4	3.5
Soybean oil	2	3.5
Di-Calcium Phosphate	1.55	1.55
Oyster shell	1.03	1.18
DL-methionine	0.01	0.01
Vitamin premix *	0.5	0.5
Mineral premix **	0.5	0.5
Salt	0.26	0.26
Sand	0.75	0.75
**Nutritional content**		
ME (KJ/kg)	2910	3030
Crude protein (%)	20.1	19
Crude fat (%)	4.6	6.14
Ca (%)	0.95	0.9
Total *p* (%)	1.23	1.06
Available *p* (%)	0.45	0.36
Methionine (%)	0.5	0.38
Lysine (%)	1.01	1
Methionine + Cysteine (%)	0.83	0.71

* vitamin A, 3,600,000 IU; D3, 800,000 IU; vitamin E, 7200 IU; vitamin B1, 710 mg; vitamin B2, 2640 mg; vitamin B6, 1176 mg; vitamin B9, 400 mg; vitamin B12, 6 mg; vitamin k3, 800 mg; pantothenic acid, 3920 mg; ; vitamin Biotin, 40 mg; vitamin Niacin, 12,000 mg and choline chloride, 200,000 mg. ** Mn, 40,000 mg; Fe, 20,000 mg; Zn, 33,900 mg; Cu, 4000 mg; I, 400 mg and Se, 80 mg.

**Table 2:** Effect of experimental diets on performance parameters of broiler chickens.

Performance Parameters	Dietary Treatments					Standard Error of the Mean (SEM)	*p*-Value
	Control	Probiotics	*Z. officinale* Roscoe				
			0.15%	0.20%	0.25%		
Feed intake (g/chick)							
1–21 days	1195 ^b^	1222 ^a^	1207 ^ab^	1187 ^bc^	1163 ^c^	8.3	0.005
22–42 days	3384	3145	3350	3597	3235	163.3	0.775
1–42 days	4578.7	4367	4558	4584	4397	162.2	0.787
Weight gain (g/chick)							
1–21 days	740	773	765	759	768	7.6	0.080
22–42 days	1321	1100	1379	1340	1197	112.6	0.434
1–42 days	2061	1873	2144	2090	1965	117.8	0.532
Feed conversion ratio (g/g)							
1–21 days	1.61	1.57	1.59	1.56	1.51	0.022	0.090
22–42 days	2.56	2.90	2.44	2.55	2.76	0.158	0.325
1–42 days	2.22	2.34	2.13	2.19	2.25	0.073	0.418

Means within the same row with different superscripts differ significantly (*p* < 0.05). ^a–c^: means within the same row with different superscripts differ significantly (*p* < 0.05).

**Table 3:** Effects of experimental diets on broiler carcass components (g).

Item	Dietary Treatments					SEM	*p*-Value
	Control	Probiotics	*Z. officinale* Roscoe				
			0.15%	0.20%	0.25%		
Carcass weight	1564	1443	1708	1647	1510	96.1	0.365
Empty carcass weight	1298	1192	1437	1362	1510	79.7	0.285
Breast	452	409	497	462	448	28.8	0.389
Thigh	425	393	458	445	417	22.6	0.351
Wings	117	117	130	126	111	7.1	0.385
Liver	60	44	51	48	44	3.6	0.108
Gizzard	68 ^a^	51 ^b^	55 ^b^	55 ^b^	51 ^b^	4.0	0.037
Abdominal fat	47 ^a^	27 ^b^	29 ^b^	27 ^a^	25 ^b^	10.0	0.029

^a,b^: means within the same row with different superscripts differ significantly (*p* < 0.05).

**Table 4:** Effect of experimental diets on serum biochemical parameters of broilers at 42 days of age.

Item	Dietary Treatments					SEM	*p*-Value
	Control	Probiotics	*Z. officinale* Roscoe				
			0.15%	0.20%	0.25%		
Total protein *	4.41	4.58	4.51	4.09	4.60	0.35	0.841
Albumin *	2.30	1.68	1.78	1.79	1.98	0.226	0.371
Glucose **	207.41	209.15	207.19	205.45	208.71	14.594	0.939
Cholesterol **	138.18	143.43	152.73	138.18	145.05	8.17	0.710
Triglyceride **	47.19	26.97	111.61	26.96	30.71	13.76	0.155
HDL-cholesterol **	63.33	78.67	70.67	69.67	79.67	7.613	0.710
LDL-cholesterol **	71.58	59.37	59.74	63.12	49.08	13.66	0.837
VLDL-cholesterol **	9.43	5.39	22.32	5.39	6.14	0.986	0.155
Uric acid **	5.41	3.68	3.54	5.00	5.27	0.79	0.309
Alkaline phosphatase ***	1463.3	1333.5	1426.7	1476.7	1320.0	1473.8	0.905

* (g/dL); ** (mg/dL); *** (IU/dL).

**Table 5:** Effect of experimental diets on immunity parameters of broilers (log_2_).

Item	Dietary Treatments					SEM	*p*-Value
	Control	Probiotics	*Z. officinale* Roscoe				
			0.15%	0.20%	0.25%		
Antibody response to Newcastle disease virus (NDV) vaccine							
Day-28	4.00	4.66	3.00	3.33	2.33	0.51	0.07
Day-42	2.33	2.33	1.33	1.66	2.0	0.29	0.152
Antibody response to avian influenza (AI) vaccine							
Day-28	1.66	2.33	1.00	1.66	1.33	0.47	0.408
Day-42	0.33	1.33	1.00	1.00	1.33	0.44	0.533
Ig M antibody *							
7 days after 1st injection (day-28)	2.33	4.66	4.66	4.00	6.00	0.89	0.139
7 days after 2nd injection (day-42)	4.33 ^a^	6.00 ^ab^	4.33 ^a^	5.33 ^a^	5.33 ^a^	0.64	0.355
Ig G antibody *							
7 days after 1st injection (day-28)	1.00	2.33	2.00	2.33	3.66	0.42	0.056
7 days after 2nd injection (day-42)	2.66 ^a^	3.33 ^a^	2.66 ^a^	3.33 ^a^	3.33 ^a^	0.66	0.736
Total antibody *							
7 days after 1st injection (day-28)	1.33	2.33	2.66	1.66	3.33	0.49	0.101
7 days after 2nd injection (day-42)	1.66	2.66	2.00	2.00	2.00	0.33	0.736

* Immunoglobulin antibody titres against sheep red blood cell (SRBC). ^a,b^: means within the same row with different superscripts differ significantly (*p* < 0.05).

**Table 6:** Effect of experimental diets on ileum microbiota (log_10_ CFU/g) of broilers.

Treatments	*Escherichia coli*	Coliform Bacteria	*Lactobacillus* sp.	Total Aerobic Bacteria
Control	5.25	6.5	7.43	8.15
Probiotics	6.34	7.91	7.45	7.43
0.15% *Z. officinale Roscoe*	6.74	8	6.62	7.66
0.20% *Z. officinale Roscoe*	6.98	8.07	7.8	7.72
0.25% *Z. officinale Roscoe*	6.21	6.97	7.95	7.35
SEM	0.669	0.49	0.339	0.353
*p*-value	0.204	0.113	0.997	0.319
